# Get Your Paws off of My Pixels: Personal Identity and Avatars as Self

**DOI:** 10.2196/jmir.1299

**Published:** 2010-07-13

**Authors:** Mark Alan Graber, Abraham David Graber

**Affiliations:** ^3^University of IowaDepartment of PhilosophyIowa CityUSA; ^2^University of IowaCarver College of MedicineDepartment of Family MedicineIowa CityUSA; ^1^University of IowaCarver College of MedicineDepartment of Emergency MedicineIowa CityUSA

**Keywords:** Autonomy, rights, avatar, virtual world

## Abstract

There is an astounding silence in the peer-reviewed literature regarding what rights a person ought to expect to retain when being represented by an avatar rather than a biological body. Before one can have meaningful ethical discussions about informed consent in virtual worlds, avatar bodily integrity, and so on, the status of avatars vis-à-vis the self must first be decided. We argue that as another manifestation of the individual, an individual’s avatar should have rights analogous to those of a biological body. Our strategy will be to show that (1) possessing a physical body is not a necessary condition for possessing rights; (2) rights are already extended to representations of a person to which no biological consciousness is attached; and (3) when imbued with intentionality, some prostheses become “self.” We will then argue that avatars meet all of the conditions necessary to be protected by rights similar to those enjoyed by a biological body. The structure of our argument will take the form of a conditional. We will argue that *if* a user considers an avatar an extension of the self, *then* the avatar has rights analogous to the rights of the user. Finally, we will discuss and resolve some of the objections to our position including conflicts that may arise when more than one individual considers an avatar to be part of the self.

## Introduction

Medical ethics is a fluid field that is constantly evolving in response to new technological developments. One of the recent innovations to impact medical ethics is the Internet and the development of virtual worlds and interactions. Virtual interactions can be as mundane as a virtual business meeting or as provocative as the Red Light Center (www.redlightcenter.com) dedicated to sex. Some virtual worlds such as Second Life (secondlife.com) allow for a range of interactions between individuals similar to those in the nonvirtual world. Among these interactions are those relevant to bioethics, particularly the treatment of various psychological disorders including group therapy, patient education, medical education, research, and so on [1-6].

An individual navigates many of these virtual worlds as a construct known as an ”avatar,” named after the worldly incarnations of the Hindu gods. Avatars may or may not share physical similarities with the person they represent. Depending on the world, avatars can be individualized by “virtual plastic surgery” to emphasize the idealized characteristics of an individual.

If virtual sex is available, can virtual rape be far behind? In the first six months of 2003, the South Korean police received 22,000 reports of crimes committed by characters in online games [7]. In 2007, the Dutch police arrested a 17-year-old, accusing him of having stolen 4000 Euros worth of virtual furniture [8], and in 2007, Belgian police opened an investigation into a man accused of virtual rape and began patrolling Second Life to prevent crimes [9].

Accusations of virtual crime have forced governments to consider the legal status of virtual property. Given that virtual property often has value in the nonvirtual world, it comes as no surprise that theft of virtual property has been prosecuted as if it were theft of material property. What does come as a surprise is the astounding silence in the peer-reviewed ethics literature regarding what rights a person should expect to retain when being represented by an avatar rather than a biological body. This lack of peer-reviewed literature is particularly surprising given the claim in the existing literature that “[i]t appears likely that [online] gaming and its associated notions of play may become a metaphor for a range of human social interactions, with the potential for new freedoms and creativity as well as new oppressions and inequality” [10]. Before one can have meaningful ethical discussions about virtual assault, virtual rape, virtual consent, and so on, the status of avatars vis-à-vis the self must first be decided.

The ability to experience virtual worlds as an avatar, along with the ability of others to experience an individual as *nothing but* an avatar, requires an examination of the paradigm of what it means to be “self,” or, to use the terminology of the philosophy literature, what it is that constitutes one’s personal identity [11]. An individual can now have more than one manifestation of a single consciousness. As new “worlds” are defined, the definition of “self” must, and will, change. This article will defend the thesis that avatars can be thought of as part of the “self” and as such have rights analogous to the rights possessed by an individual’s body in the nonvirtual world.

Our strategy will be to show that (1) possessing a body is not a necessary condition for possessing rights; (2) rights are already extended to representations of a person to which no biological consciousness is attached; and (3) when imbued with intentionality, some prostheses become “self.” We will then argue that avatars meet all of the conditions necessary to be part of “self” and thus should be protected by similar rights as a biological body. Finally, we will discuss and resolve some of the objections to our position as well as conflicts that may arise when more than one individual considers an avatar to be part of the self.

We will argue that *if* a user considers an avatar an extension of the self, *then* their avatar has rights analogous to the rights of the user. It is not our intention to argue that the majority of individuals currently consider their avatar as part of the self. Even if this isn’t currently the case, evidence suggests that with advances in virtual interface technology, more and more users will come to identify their avatar as an extension of themselves [12,13].

Throughout this paper we will use the term “nonvirtual world” to refer to our corporeal existence instead of the term “real world.” The term “real world” connotes that other worlds are not “real.” This creates a psychological bias minimizing the “realness” of virtual worlds.

## Arguments

### A Physical Body is Not Necessary for Legal Protection and Having Rights as a Person

The legal definition of psychological abuse provides evidence that one need not have a body in the usual sense to have legal rights. According to the US Department of Health and Human Services Children’s Bureau, 48 US states have laws against the emotional abuse of a dependent child. They go on to say that the “[t]ypical language used in these definitions [of emotional abuse] is injury to the psychological capacity or emotional stability of a child” [14]. In Virginia, for example, emotional abuse can take the form of “ridicule, rejection, intimidation, ignoring a child, or indifference” [15]. Even though in the nonvirtual world a consciousness is always tied to a body, a physical presence (or body) is not necessary for any of these to take place. In fact, one can easily envision this type of crime occurring in a chat room, over the phone, via email or even in a particularly scathing viral video. Thus, while a consciousness is required for this crime to take place, a corporeal body is not.

If the previous example is not convincing, a will provides more direct evidence that having a physical body is not a necessary condition for possessing rights. A will provides instructions regarding a deceased individual’s property. Having died, the author of the will no longer has a physical body nor any kind of mental activity. Even so, a person’s wishes in regard to his or her property are binding; that is to say, individuals retain some amount of postmortem property rights.

In summary, a body is not a necessary condition to possess rights. And some rights continue even when a consciousness is no longer present. This does not imply that all inanimate objects qualify for rights. But in the proper circumstances, which we will discuss below, an avatar, which has neither a corporeal existence in the nonvirtual world nor inherent consciousness, can be a candidate for rights based on being part of the “self.”

### Rights are Already Afforded to Representations of an Individual

We already afford rights to some representations of an individual that are not connected in a biological sense to the individual’s consciousness. Consider the case of an individual with prosthetic limbs. If these limbs are stolen from a house and destroyed, they are considered property: a burglary and destruction of property have occurred. Now consider two cases in which an individual is attacked. In the first attack (A1), the assailant misses the intended target entirely. This is unpleasant because of awareness of potential danger, but there is no bodily harm: the victim sustained no physical damage. In the second attack (A2), assume that the only damage that occurs during the assault is to the victim’s prosthetic limbs while they are attached to the individual. Most readers will intuit that A2 reaches a different level of harm than A1 even though the prosthetic limbs are a nonbiological manifestation of the individual*.* Some moral/legal change occurs when the prosthetic limbs are attached to the victim of the assault. Imbuing the limbs with intentionality changes the moral status of the limbs from that of inanimate objects to extensions of the individual to whom they are attached.

Perhaps a more intuitive example comes from the transplantation of a biological “prosthesis” from one person to another. Take for example, a biological face, hand, or arm graft. These are prostheses, albeit of a sophisticated nature. Is it the case that these cannot be a part of the person because they are prostheses and do not contain the same DNA? Does being part of the “self” require a prosthesis to contain the same DNA as the user? We do not believe this to be the case. Rather, the person receiving the transplanted part gives it intentionality. Otherwise the part would simply be an inanimate object, unattached, and a candidate for burial or cremation.

We would argue that a biological prosthesis does not differ fundamentally from a sophisticated mechanical arm or a functional prosthetic mechanical eye. The difference between a biological prosthetic arm and a nonbiological prosthetic arm is one of degree and not of kind. Both are prostheses and devoid of rights (as a person) unless given intentionality. As future nonbiological prostheses become more sophisticated and integrated into the nervous system, this will become more self-evident. [16,17].

It does not appear that biological identity is necessary for something to be given intentionality and thus to become self. This does not imply that every object can be considered self. However, it allows for, although does not prove, that avatars can be “self.” Avatars are analogous to prostheses in that they allow the user to manipulate the environment even though they have no direct biological connection to the consciousness of the individual. The individual thinks, moves a muscle, and the prosthesis moves. This is the same path taken by an avatar. The individual thinks, moves muscles (in this case controlling a keyboard or a mouse), and the avatar carries out an action. If, as we argue, we are going to assign “self” to one biological or nonbiological corporeal manifestation of an individual (prosthetic limbs) there should be no barrier to assigning similar rights to another nonbiological manifestations of an individual (the avatar).

Likewise, we already assign rights to the corporeal representation of an individual whether or not a biological consciousness is present *within* the representation. This is the case with rights afforded those in a permanent vegetative state. There are legal protections against assaulting and otherwise violating those in a persistent vegetative state. For our purposes, the reason for these rights is immaterial. Whether it is because there was once a consciousness present or because society finds violation of a biologically live body abhorrent is irrelevant. The point remains that consciousness need not be biologically present for the *representation* to have rights as a person. This is analogous to an avatar. It is a representation of an individual, and we will argue below that it is part of the self, even though there is no biological consciousness present within the representation.

### Avatars are Appropriate Candidates for Rights as Part of the “Self”

The fact that having a physical body is not a necessary condition for an entity to possess rights does not show that avatars have rights. A young child’s imaginary play friend does not have a physical body. It would be absurd to argue that it had rights as a person.

Our first argument showing that avatars are appropriate candidates for rights is a thought experiment. Let us imagine a “Matrix-like” world. Let us call this world MW_1_. In this world, people are directly and permanently connected to a virtual world via brain-scanning hardware. The person thinks and their avatar in the virtual world moves. The nonvirtual world and virtual worlds are phenomenologically indistinguishable. All representations of the “self” in this world are via an avatar. What happens to the avatar is fed back into the brain scan system causing pain, pleasure, and other sensations. Higher emotions such as fear, greed, and love are also affected by the virtual world. It is unquestionably true that residents of this Matrix-like world can make legitimate rights claims on each other, the right to be free from torture, for example. Any rights claimed would be assigned to the avatar since it is the only manifestation of self that can act that exists in this world.

Now let us suppose that instead of being permanently hardwired into the virtual world, the residents of MW_1_ can log in and out of the virtual world at will. Let us call this world MW_2_. Does the ability to log in and out change legitimacy of rights claims in the virtual world? Nothing of moral (and presumably legal) importance has changed about an individual’s relationship to the virtual world except that there are now two worlds in which an individual can make rights claims: a virtual world and a nonvirtual world.

Let us examine a third world, W. It is similar to MW_2_ in all ways except that brain-scan technology has yet to be invented. People can only interact with the virtual world via a computer monitor and keyboard (one may note that W looks very much like the nonvirtual world we inhabit). There is an important difference between MW_2_ and W. The behavior of, and toward, avatars can no longer cause certain phenomenological states such as physical pain or the sensations of touch, taste, and smell, for example (although hardware that allows some of these physical sensations has been developed). Other phenomenological states will still be affected by events in the virtual world such as joy, despair, anxiety, and annoyance.

The difference between MW_1_ and W is one of degree and not of kind. If the difference is only in degree, people can still make legitimate rights claims for their avatars; all that changes is the degree of strength of said claims [18]. The use of keyboard and mouse certainly adds an additional barrier between a user and his or her avatar. Though the connection between user and avatar is less direct, the intentional relationship between user and virtual construct remains. The scope of rights claims is all that differs.

### Degree of Identity and Rights

Given that an action that would cause physical pain to the user in MW_2_ will cause no such pain for a user in W, some behavior that is not permissible in MW_2_ will be permissible in W. Just as one has a right not to be assaulted, even when the assault causes no physical harm, avatars in W will have certain rights as part of the self. These may include bodily integrity and freedom from fear. The closer the identification between the avatar and the individual, the more complete the rights of the person-as-avatar. In the “Matrix-like” world, where phenomenologically the prosthetic is the person, the rights of the avatar are identical to those of the person

Starting with a paradigm that made the case for legitimate rights claims in MW_1_, we made modifications that made MW_1_ come to resemble W, which is in all relevant aspects similar to our own world. We further argued that each modification was only a change of degree, and not a change in kind. Given that avatars in MW_1_ have rights, it follows that avatars in W also have rights.

Our final argument in favor of the rights of avatars is related to the previous argument, that is, avatars have rights by proxy of the rights of their users*.* For example, while my avatar is engaged in an online community I find myself facing unwelcome sexual advances. Things quickly escalate out of control. I find myself feeling extremely uncomfortable. Depending on the situation, my only recourse may be to disconnect my avatar. This looks like a violation of right to free access. One may now argue that “Aha, didn’t they just say the rights of an avatar are dependent on the proxy rights of the individual and not inherent to the avatar?” To this we would answer an emphatic yes. It shows the degree of identity between an avatar and the ”person.” Essentially, they are phenomenologically one and the same at this point. Changing the rights of the avatar changes the rights of the person. My avatar has certain rights and violating those rights is also a violation of my rights.

### “Normal” Interactions and “Necessary Embeddedness”

The ability of a person to have interactions in more than one social milieu (virtual and nonvirtual) requires a redefinition of the “self.” One way we propose to redefine self is by using a principle we call “necessary embeddedness.” An object is “necessarily embedded” and has rights as part of the self to the extent that the object is necessary for “normal” interaction within some specific domain. Note that “normal interactions*”* change as the domain changes. What counts as a normal interaction in the nonvirtual world is not a paradigm of normal interaction in the virtual world. Additionally, what count as “normal” interactions in the virtual world can and do change as technology and programming evolve, allowing ever more phenomenologically complex interactions to occur. By basing the standard for rights possession by nonbiological extensions on “normal” interactions*,* necessary embeddedness captures the social nature of rights and manages to apply them to inanimate objects. For example, persons who are visually impaired may use haptic devices to provide tactile feedback in lieu of visual cues. In the case of the vitual world, haptic devices can substitute for vision allowing people who are visually impaired to locate other online individuals, navigate passageways, and so on [19]. Without such a device, the virtual world is essentially inaccessible to people who are visually impaired [20]. Thus, we argue that the haptic device would enjoy protection as part of the self should the user wish to deem it so. For any individual reliant on it, the haptic device is so fundamentally a part of the self within the virtual environment that removing it would change the “self” vis-à-vis that particular world. Likewise, although the avatar is not material, it is just as fundamental as a haptic device for interactions in virtual worlds.

We would also argue that applying the principle of necessary embeddedness may correct our mistaken intuitions on rights. In our Matrix thought experiment, it is not intuitively clear that avatars in W have rights; however, it is clear that avatars in MW_1_ have rights. We posit that because avatars in MW_1_ are more strongly “necessarily embedded” than those in W, our intuitions recognize the legitimacy of rights claims in MW_1_ while they do not for avatars in W.

### Response to Possible Objections

By this point the observant reader will have noticed a somewhat disconcerting trend with our argumentation. Any number of our arguments seem to lead to a question with an unacceptable answer: If an individual decides that a doll (or other object) is his representation in the nonvirtual world, should it have rights as “self?” By the argumentation expressed so far, it would appear that we must also accept the conclusion that a doll is an appropriate candidate for rights if an individual deems it so.

We have two responses to this conclusion. First, we intuitively assign rights to prosthetic limbs. As demonstrated above, we recognize on some level that prostheses are part of “self” when attached to an individual. Prostheses in the future will be more integrated with “self” as prostheses that interface directly with the brain and the biological body are developed [17,18]. Second, we would argue that the “self” is defined in a positive sense (at least in part) as that which allows an individual to interact with the world in a “normal” fashion. And, in a negative sense, if this “self” is disturbed somehow (prosthetic limbs are removed from the individual, or an avatar is attacked and disabled) its ability to interact in the world is hindered. Neither of these conditions is met by a doll.

This still begs the point about assigning “self” to a doll or a pair of glasses. We would suggest that if someone posits that “a doll is my representation in the world and part of my “self,”” it has to meet the “test of destruction,” that is, would destruction of this doll hinder my ability to function in the world in a fundamental sense (not just the depression suffered from the loss of an object)? Does the doll meet the test of necessary embeddedness? If the answer is no, it is not considered “self.” It is clearly separate from self in that its destruction does not fundamentally change the individual’s ability to function in the world. Similarly, one can raise the question about glasses, a wheelchair, or the white cane carried by visually impaired persons. We would argue that to the extent that individuals imbue these prostheses with intentionality and they are needed for “normal” interactions (necessarily embedded*)* with the world, they should be recognized as an extension of one’s self. Thus, they have a legitimate right to protection while in use; interfering with their active use would constitute assault and not a property crime. An analogy would be plucking out a functional prosthetic eye or a cochlear implant. As in the Matrix scenario, there is a difference in degree between interfering with someone’s white cane and their prosthetic eye but not a difference in kind.

There may be a finite number of situations (eg, psychosis, delusions, or delirium) in which an individual does assign “self” to a doll, and the doll’s destruction hinders or prevents the individual from interacting in the world (the doll is necessarily embedded in the individual’s interactions). In this case, we would argue that the doll does have a right to bodily integrity as part of the self of the individual; attacking the doll has a fundamental consequence for the individual. However, this would apply to a very limited subset of individuals. Another caveat is that one obviously cannot define another individual as “self” even if the other “self” is being used as a prosthesis (for example, someone who is helping you cross the street). Nor can one claim as “self” a prosthesis to which someone already has a right. This is discussed further below.

### Identity and Conflict

As we note above, rights require a social milieu. Thus, conflicts can arise which require rules. An interesting case occurs when more than one person claims a prosthesis as part of the self. For example, imagine a team of scientists who work with the Mars Rover. The Mars Rover is necessarily embedded for the scientists; it is the only way for them to interact with the surface of Mars. Some particular scientist (perhaps a bit deluded) comes to think about the Mars Rover as a part of himself. The Mars Rover then fulfills the two criteria for rights that we laid out previously: it is necessarily embedded and it is viewed as part of the self. Now imagine that not one but two (slightly deluded) scientists claim the Mars Rover as an extension of the self. Is the Mars Rover an extension of both scientists’ selves? There are two caveats to add to the theory of necessary embeddedness. First, if an agent, A, is aware that some object, O, is possessed by another individual, A cannot extend rights to O. A’s knowledge of the preexisting rights claim by some other person precludes A’s making it a legitimate extension of the self. In the case of the Mars Rover, the scientists cannot extend their selves to the Rover because the Rover is known to be public property. Second, if two individuals make two competing, seemingly equal legitimate rights claims on an object at the same time, property law would come into play to resolve the conflict. If the claims are indeed equal, the claims would cancel each other out and neither scientist would be able to claim the object as self.

A more interesting scenario occurs when “borrowing” a prosthesis. Imagine a world similar to MW_1_; call it MW_1_* (recall that MW_1_ is the “Matrix-like” world). In this world, two individuals share the same avatar. When one person goes to sleep, the other person wakes up and controls the avatar. In this case there does not seem to be any ownership claims that might undermine one individual’s rights claims to the avatar as self. Nor is it the case that the rights claims overlap temporally; each individual makes his or her rights claim in turn *and with the agreement of the other.*

The concept of something serially being the “self” of two individuals seems at first counterintuitive. However, a real-world example would be a transplanted heart. The heart is serially part of the “self” of two individuals. In fact, if you remove it, the “self” of each individual will cease to exist (short of technological support). And, there is no inherent barrier to the heart being transplanted serially to others (once immunologic barriers are removed). Thus, the concept of something being the “self” of two individuals serially does not require a paradigm shift. In the case of the avatar, if there is an intention to deceive other people (for example identify theft) on the part of one party using the avatar, laws about personal identity come into play.

If the two individuals do not know each other and are not aware they are sharing an avatar, then there is a rights conflict (although from a practical perspective, by definition, it remains undiscovered). This would be similar to someone living in my vacation home without my knowledge. The fact that I am unaware of the occurrence does not mean that I do not have rights to my property.

### The Right to Bodily Integrity

The right to bodily integrity is *one* of the most fundamental rights. Thus, it should be afforded to avatars in that they can be considered part of “self.” The degree of rights assigned to an avatar as part of the self will depend on the degree of identity between the avatar and the person. In MW_1_ (the “Matrix-like” world), the identity between the avatar and the self is complete. Thus, the avatar, as the only social and phenomenological manifestation of self, will be entitled to the full rights enjoyed by a person (sans rights to such things as health care that may not be not applicable).

We believe it is obvious a priori that the right to bodily integrity is one of the most fundamental rights. Before one can even contemplate higher rights (to free speech, to own property, etc), one needs to be free from fear of assault, rape (a particularly heinous type of assault), torture, murder, and so on. As noted above, certain states—such as joy, despair, anxiety, annoyance, and love—may be felt as direct consequences of *current* virtual interactions. Thus, assault or other violation of the self-as-avatar will have consequences in the nonvirtual world. The likelihood of this will increase in the future as more complex tactile feedback is given to the user (pain, pleasure, etc).

That avatars are candidates for rights also raises the question of informed consent within virtual worlds. We would argue that some type of informed consent should be required for virtual world research. Even a research questionnaire will have an impact on the individual whether the questionnaire is administered in the virtual or nonvirtual world. The virtual world inherently affords a degree of anonymity that may or may not exist in the nonvirtual world depending on the study design (eg, whether the questionnaire is administered in person). However, there will be some emotional impact on the individual in the study. Thus, consent, perhaps in a modified form, should be obtained in the virtual world as it is in the nonvirtual world.

## Conclusion

We have outlined a case showing that avatars, as extensions of the self, are candidates for rights. The subset of rights one’s avatar should enjoy will be dependent on the degree of phenomenological identity between the individual and his or her avatar. Drawing this line is difficult. Some people will have a stronger emotional reaction to a violation of their avatar than others. And, the situation in which the avatar is participating will obviously modify these rights. One would expect a greater degree of bodily integrity within virtual group therapy than in a combat simulation.

Areas of future research could focus on expanding the discussion of necessary embeddedness and it’s application to virtual and nonvirtual worlds as well as further discussion of what elements of informed consent are necessary in a virtual world. A discussion of what might constitute the “inalienable” rights of an avatar should also be undertaken.
